# Predictors of academic performance during the covid-19 outbreak: impact of distance education on mental health, social cognition and memory abilities in an Italian university student sample

**DOI:** 10.1186/s40359-021-00649-9

**Published:** 2021-09-15

**Authors:** Laura Giusti, Silvia Mammarella, Anna Salza, Sasha Del Vecchio, Donatella Ussorio, Massimo Casacchia, Rita Roncone

**Affiliations:** 1 Department of Clinical Medicine, Public Health, Life and Environmental Science, University of L’Aquila, L’Aquila, Italy; 2 Counselling and Consultation Service for Students (SACS), University of L’Aquila, L’Aquila, Italy

**Keywords:** COVID-19 outbreak, Distance education, Mental health, Depression, Cognitive difficulties

## Abstract

**Background:**

This study aimed to investigate the impact of distance education (DE) on mental health, social cognition, and memory abilities in a sample of university students during the national COVID-19 lockdown in Italy and to identify the predictors of academic performance.

**Methods:**

Two hundred and three students (76.4% women, mean age 24.3, SD ± 4.9) responded to an anonymous online cross-sectional survey between July 15 and September 30, 2020, on DE experience and cognitive and social-cognitive variables. A short version of the Beck Depression Inventory-II, ten images from the Eyes Task, and five memory vignette stimuli were included in the survey. Descriptive, one-way ANOVA, correlation, and logistic regression analyses were conducted.

**Results:**

Half of the student sample reported significant impairment in concentration and learning abilities during DE. Regarding psychological health, 19.7%, 27.1%, and 23.6% of the sample reported mild, moderate, and severe depressive symptoms, respectively. Correlation analyses showed a statistically significant negative association between depression and the overall subjective evaluation of DE (r =  − 0.359; *p* < 0.000). Changes in one’s study context and habits, i.e., studying alone at one’s parents’ home instead of studying with colleagues or alone in a university “social place” (e.g., the university library), seemed to increase the likelihood of poor academic performance by almost 3 times (O.R. 3.918; *p* = 0.032). This predictor was no longer statistically significant in the subsequent step when the individual impairment predictors were entered. Learning concentration impairment during DE (O.R. 8.350; *p* = 0.014), anxiety about COVID-19 contagion for oneself or others (O.R. 3.363; *p* = 0.022), female gender (O.R. 3.141; *p* = 0.045), and depressive symptomatology (O.R. 1.093; *p* = 0.047) were ultimately determined to be the strongest predictors of poor academic performance, whereas the appreciation of DE represented a protective variable (O.R. 0.610; *p* < 0.000).

**Conclusions:**

The study showed a negative impact of DE on the mental health of students presenting depressive symptoms and impairment in concentration and learning, the latter identified as the strongest predictors of poor academic performances. The study confirms the emerging need to monitor the impact of DE, which occurred during the 2019/2020 academic year and will continue in the coming months, to refine educational offerings and meet students' psychological needs by implementing psychological interventions based on the modifiable variables that seem to compromise students’ psychological well-being and academic outcomes.

**Supplementary Information:**

The online version contains supplementary material available at 10.1186/s40359-021-00649-9.

## Introduction

On March 11, 2020, the World Health Organization listed the coronavirus pandemic as a Public Health Emergency of International Concern [1]. Since Italy’s COVID-19 lockdown, a range of containment measures have been urgently adopted (i.e., the closure of all schools, universities and workplaces and home confinement) to contain the spread of the infection, locking down the whole country and prohibiting people from assembling.

According to many international studies, the COVID-19 pandemic has led to high levels of psychological distress [2], depression [3], anxiety [4–6], and panic behaviours [7]. Liang et al. [8] reported that nearly 40.4% of the youths in their sample were prone to psychological problems and that 14.4% showed post-traumatic symptoms.

In the Italian context, some studies have assessed the negative psychological consequences of the pandemic on the general population, indicating that female gender, infection of an acquaintance, history of stressful situations and medical problems, and less adequate physical space during isolation, among others, to be predictive factors [9, 10]. The severe impact on the population's mental health occurred within the context of a drastic reduction in care levels in Italy [11].

The changes related to the COVID-19 outbreak have also affected the academic context. In fact, all universities have faced and are still facing many challenges [12]. Since March 2020, distance education (DE) has replaced traditional face-to-face teaching. The massive use of technologies, resulting from the necessity for social distancing due to the COVID-19 pandemic, required great flexibility from university students and teachers. Additionally, home confinement compromised the possibility of fully experiencing university life, influencing academic study (i.e., uncertainties about cancellation, delays in activities, and digital platform use) and limiting the ability to benefit from social support, which can play a crucial role in facing the difficulties of the university environment [13, 14].

A longitudinal study investigating the relationship between mental health and social networks in the university population found that COVID-19-specific worries, lack of interaction and emotional support, and physical isolation were associated with negative mental health trajectories, especially in female students [13]. An investigation of the Spanish university population reported higher anxiety and depressive symptoms in students than university staff (administrative and teaching staff), showing that students suffered most from the psychological impact of the COVID-19 health emergency [15]. The high levels of symptomatology in students seemed promoted by the uncertainty and the potential negative impact on academic progress. Another study found that students were concerned about their education, examinations, progression to the next academic year, and wellbeing and that they showed symptoms of anxiety [16]. A Chinese study reported a psychological impact of the COVID-19 pandemic on approximately 25% of college students, who showed anxiety of different severity levels that were significantly correlated with negative effects on daily life and delays in academic activities [17]. The authors identified residence in urban areas, family income stability, and residence with parents to be protective factors and infection of relatives or acquaintances with COVID-19 to be a risk factor. The stability of family income was a significant factor in students’ experienced anxiety during the COVID-19 crisis, which could be explained by increased psychological and economic pressure [17]. Living with parents was another favorable factor against feeling anxious. Relatives or acquaintances being infected with COVID-19 was an independent risk factor in college students’ anxiety about the epidemic, which might be related to the high contagiousness of the new coronavirus pneumonia [18, 19]. Other studies reported anxiety and depression symptoms in university students following the outbreak [15, 20, 21].

An Italian qualitative and quantitative study conducted with 103 university students [22] reported that 21.4% of help-seeking students experienced lockdown to be a traumatic experience, 36% of the student sample experienced psychological distress due to anxiety symptoms, and 26% showed depressive symptomatology. Additionally, the authors found that the students experienced changes in their sleeping patterns (68%), difficulty concentrating (67%), and loss of energy (58.6%). The likelihood of experiencing post-traumatic symptomatology seemed to increase by more than 3 times with the length of home confinement (two months), and an “all-or-nothing” cognitive thinking style was the final strongest predictor, increasing the risk of traumatic distress by more than 5 times [22], confirming that maladaptive appraisals can predict severity of stress reactions after a traumatic event and mediate adaptive functioning to environmental stressors [23].

A recent follow-up survey of undergraduate medical students [24] found a significant increase in anxiety and stress levels, with depression remaining unchanged during COVID-19, irrespective of gender, year of study, place of residence, and family monthly income; poor sleep quality and higher levels of baseline depression, anxiety, and stress were found to be significant predictors of negative mental health. Stress, anxiety, and depressive thoughts among students were also found in a recent study, mainly related to difficulty concentrating, disruptions to sleeping patterns, decreased social interactions due to physical distancing, and increased concerns on academic performance [25].

A relatively unexplored, interesting issue concerns students’ academic performance outcomes and their predictors related to students' psychological status and the frequently reported traumatic distress and impairment of concentration during the COVID-19 lockdown. Furthermore, in the implementation of precautionary national measures against COVID-19, DE seems to have influenced levels of psychological distress and challenging learning abilities [26–29].

The current study aimed to investigate (1) the impact of DE during the COVID-19 pandemic on the psychological health, social cognition and memory function of a sample of university students from the University of L'Aquila and (2) the predictors of academic performance during DE.

Considering the stressful conditions of the COVID-19 outbreak, we expected a relevant impact of DE and academic changes on students, including on their depressive symptoms, difficulties concentrating, and memory in social cognition; thus, we considered these variables to be possible predictors of low performance during DE.

## Methods and materials

### Context

Located in Central Italy in the town administrative centre of the Abruzzo Region, the University of L'Aquila is a public teaching and research institution offering a full range of academic programs, including in biotechnologies, sciences, economics, engineering, education, humanities, medicine, psychology, and sport sciences. With seven departments, the University of L'Aquila offers 69 degree programs (divided between first- and second-level degrees), nine research doctorate programs, specialization schools, specialized master’s programs, and vocational programs to over 19,000 enrolled students. The faculty includes approximately 600 professors and researchers.

In L'Aquila, the COVID-19 health emergency and related academic-organizational changes represented an additional challenge to face after the devasting earthquake that hit L’Aquila on April 6, 2009, bringing death and destruction to the university, with 55 students killed [30–32].

The Department of Life, Health and Environmental Science of the University of L'Aquila manages 17 study programs (7 s-level degree programs and 10 first-level degree programs) in 3 areas—medical, biological, and environmental sciences—with 2773 students enrolled in 2019 and with a teaching staff of 125 tenured teachers.

This study was conducted by the Counselling and Consultation Service for Students (SACS) of the University of L'Aquila (Italy) [33]. The SACS, established in 1991, aims to support and help students who experience difficult moments due to failure in their studies or psychological distress.

### Study design and participants

Starting on March 13, 2020, the University of L'Aquila made it possible to start DE through the use of the Microsoft Teams platform. The SACS planned an assessment study and developed the protocol of the study, including a cross-sectional online anonymous survey using a convenience sample from July 15 to September 30, 2020, before the beginning of the new 2020–2021 A.Y.

The survey was the result of an online focus group conducted on Microsoft Teams® (Microsoft Corporation, Redmond, Washington, USA) to develop concepts and questions for the questionnaire design. The focus group meeting lasted 2 h and included SACS professionals, teachers and students.

On July 15, 2020, we uploaded the questionnaire to the department's home page and advertised it as the "Studying with COVID" survey. Via the department’s page, all university students regularly enrolled in a degree program at the University of L’Aquila were invited to participate in the survey on their DE experience during the COVID-19 health emergency.

The students did not receive any form of compensation for participation in this study.

### Survey instruments and related measures

The questionnaire (Additional file 1: Appendix 1) consisted of three sections.

Section 1 included information on the study, protection of privacy and informed consent.

In Section 2, the students were asked questions about demographic and academic data and the experience of DE, including on the following topic:

- Delivery of DE, including connections, availability of digital devices, use of the Teams platform, problems with overlap in the family environment about the use of WiFi networks and hardware;
- Academic learning, including learning concentration impairment and possible factors involved in decreased learning concentration, study context and habits after the pandemic, factors influencing exam preparation during the lockdown, and impact on exam outcomes;
- Student self-assessment of overall academic performance;
- Advantages and disadvantages of DE, including the accuracy of lessons, downloading of recorded lessons, respect of lesson time, contact with teachers outside class hours, a “sense of a team” between students and teachers, reduction in travel time to reach the university, lessons without face-to-face contact with teachers and other students, lack of interaction and difficulties contacting teachers, difficulty with specific teaching methods and difficulty attending professional laboratories and internships;
- Overall evaluation of the DE experience on a 10-point Likert scale (1 = not at all satisfied; 10 = very satisfied).

Section 3 section included psychopathological, cognitive and social cognition measures. To investigate the well-being levels of the students, 10 items from the Beck Depression Inventory II (BDI-II) [34] Italian validation [35], scale were included. The BDI-II consists of 21 items based on the DSM-IV-TR criteria and is the most frequently used tool to assess the presence and severity level of depressive symptoms. In the current study, 10 items were selected to facilitate the completion of the tool. Specifically, the following items were selected: item 1 (sadness), item 2 (pessimism), item 4 (loss of pleasure), item 12 (loss of interest), item 15 (loss of energy), item 16 (sleep), item 17 (irritability), item 18 (appetite), item 19 (concentration), and item 20 (fatigue). In the original BDI-II, each item is rated on a 4-point Likert scale (0–3). Higher scores correspond to greater psychopathological impairment. A score of 0–13 indicates an absence of depressive symptoms, a score of 14–19 indicates mild depression, a score of 20–28 indicates moderate depression, and a score of 29–63 indicates severe depression. In our adaptation of the original scale, based on the selected items and a total score of 30, we set a cut-off score of 6 to indicate the presence of depression, with scores from 6 to 8 indicating mild depression, scores of 9–12 indicating moderate depression, and scores of 13–27 indicating severe depression.

The Eyes Task (a revised version of the Reading the Mind in the Eyes Test) [36], Italian validation [37], is an advanced theory of mind task that assesses the ability to infer complex mental and emotional states through the decoding of eyes. Participants are presented with a series of 36 photographs portraying the eye and face region of a variety of individuals. Each photo is presented with four potential complex mental states from which the participant chooses. The participant must choose which feeling or thought best describes the subject’s mental state. The score has a range of 0–36: a high score indicates a good ability to decode eyes and attribute mental states to them. For the current survey, 10 items were selected that depicted the eyes of both female and male faces. A score ranging from 0 to 10 was considered, with one point for each correct answer.

To assess attention and memory abilities, five vignette stimuli taken from Module 5, “Over-confidence in memory errors,” of the metacognitive training (MCT) intervention [38, 39] were used; this module was designed to improved memory abilities and reduce over-confidence in memory errors. The student was invited to linger no more than ten seconds on the five selected images representing scenes of everyday life depicted according to common and socially shared frames and scripts. The total score ranged from 0 to 5, with one point for each correct answer.

At the end of the questionnaire form, each student was asked to evaluate his/her emotional condition perceived after the period of social confinement ("C*ompared to the beginning of last May (end of confinement), how would you judge your emotional condition now*?") with a scale of 5 answers (5 = Much better now; 4 = A little better now; 3 = More or less the same; 2 = A little worse now; 1 = Much worse now).

The current study was approved by the Internal Review Board Committee of the University of L’Aquila.

### Statistical analyses

Statistical analyses were conducted in four phases: (1) descriptive analysis of socio-demographic, academic data and subjective evaluations of DE and related aspects; (2) one-way ANOVA and chi-square tests to examine differences in socio-demographic variables and differences in the variables related to aspects of DE among students based on gender, degree program and year in the program; (3) correlation analysis (Pearson’s r) to examine relationships among the overall evaluation of the DE experience, age of participants, depressive symptoms and cognitive and socio-cognitive variables; and (4) logistic regression analyses.

Regression analyses were conducted to identify potential predictors of low academic performance during the COVID-19 lockdown. The students’ self-evaluations of their overall study performance during DE were coded into two categories: high (students reporting no problems, only occasional difficulties, or some difficulties) and low (many difficulties or major difficulties).

Logistic regression was used to test one predictive model. We included three blocks of variables. In step 1, socio-demographic factors (gender, age group, and working student) were included as potential predictors. Age was coded into two categories (19–25 years = 1 and 26 years and above = 0). This categorization was based on the assumption that women and younger people might be more at risk for developing distress impacting their academic performance. Working student was coded into two categories (no = 0/yes = 1). In step 2, related to the main consequences of students’ social isolation experience during DE, we included as potential predictors a lack of interaction and sharing experiences with other students and a transition from a “social” study setting to the setting of one parents’ home. Both predictors were coded into 2 categories (no, items scored 1–3 indicating no difficulties, occasional difficulties, or some difficulties/yes = 0, items scored 4–5 indicating many difficulties or major difficulties = 1). In step 3, we included predictors related to the participants’ cognitive, social cognition, memory, and psychopathological impairment (COVID-19 contagion anxiety and depressive symptoms) and global satisfaction with DE, assuming that all these variables could have a relevant impact on study performance.

Learning concentration impairment during DE was coded into 2 categories (no, students referring to no difference or improvement of their abilities = 0/yes, students referring to a worsening of their abilities = 1). The Eyes Task score was based on the total 10-item scale score and coded in two categories (accurate, Eyes Task scores of 4–10 = 0/inaccurate, Eyes Task scores of ≤ 3 = 1). COVID-19 contagion anxiety for oneself or others was coded in two categories (no, item scores of 1–3 = 0/yes, item scores of 4–5 = 1). The memory task score, the 10-item BDI-II total scores, and the DE global evaluation score were entered as continuous variables.

Statistical analyses were conducted using SPSS 27.0 (SPSS Inc., Chicago, IL, USA).

## Results

### Descriptive analyses

A total of 203 students participated in our survey, of whom 155 were women (76.4%) and 48 were men (23.6%); the mean age was 24.3 years (SD ± 4.9), with no significant gender difference in age. The registered mean duration of questionnaire completion was 22 min and 52 s.

The socio-demographic data of the student sample are reported in Table 1. More than 50% of the students in our sample were medical students, followed by almost 15% of students in psychiatric rehabilitation techniques. Of the 17 programs of the Department of Clinical Medicine, Public Health, Life and Environmental Science, the students who participated in this survey were enrolled in 12. Almost 20% were students taking longer than normal to complete their program, and more than 80% were off-site students. Of our sample, 16.7% are student workers. The survey was mainly taken by students enrolled in their second year (30.5%), followed by freshmen students, who represented 20.2% of the sample, showing that these two students more actively participated in the survey than other groups.

**Table 1:** Socio-demographic data of the student participants in the survey (n = 203)

	Total (%)
*Gender*	
Men	48 (23.6)
Women	155 (76.4)
*Age, mean*	24.3 (SD ± 4.9)
*Degree programs*	
Medicine and surgery	104 (51.2)
Dentistry	7 (3.4)
Psychiatric rehabilitation techniques	29 (14.3)
Nursing	15 (7.4)
Obstetrics	2 (1)
Speech therapy	3 (1.5)
Neuro-psychomotor therapy in developmental age	5 (2.5)
Prevention techniques for the environment and the workplace	10 (4.9)
Orthoptics	1 (0.5)
Master's program for health professions	5 (2.5)
Biology	12 (5.9)
Master's program in biology	10 (4.9)
*Academic year*	
Students who take longer than normal to complete their program	37 (18.2)
Freshman	41 (20.2)
Second year	62 (30.5)
Third year	35 (17.3)
Fourth year	11 (5.4)
Fifth year	9 (4.5)
Sixth year	8 (3.9)
*Off-site students*	166 (81.8)
*Working students*	34 (16.7)

### The impact of DE during the COVID-19 pandemic on the psychological health, social cognition and memory function

**Fig. 1:**
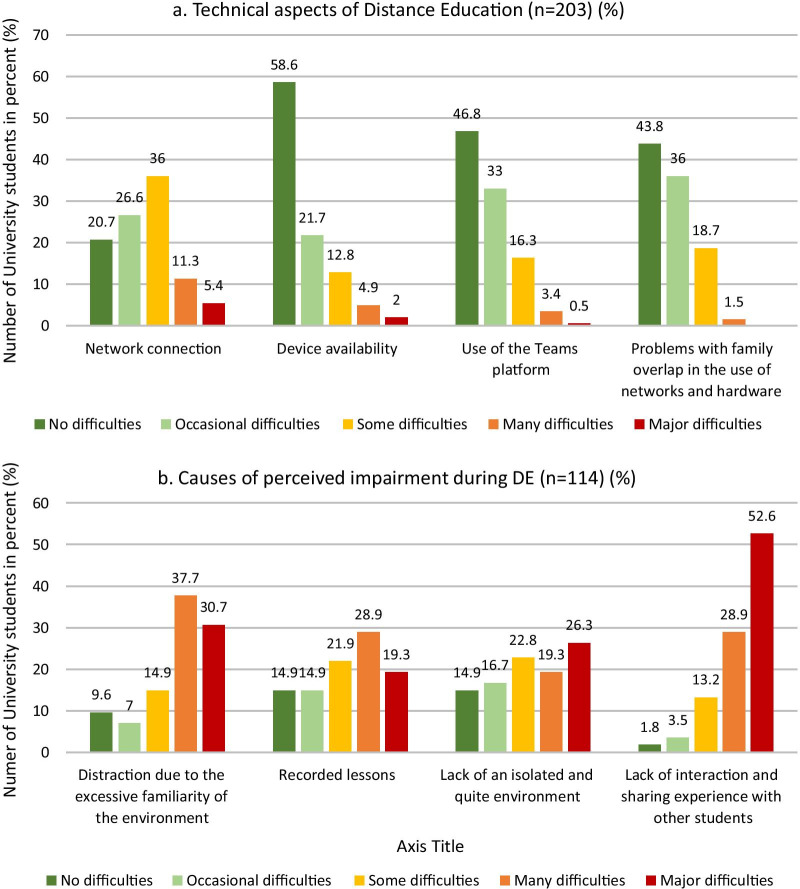
**a** Response rates of the interviewed students regarding technical difficulties encountered during DE (n = 203). **b** Student response rates identifying possible factors involved in worsening learning concentration attending DE (n = 114)

Regarding the technical difficulties perceived by students during DE (Fig. 1a), the students mainly attributed many or major difficulties to the need to share a Wi-Fi network with other family members or friends (20.2%) and to network connection problems (16.7%).

**Fig. 2:**
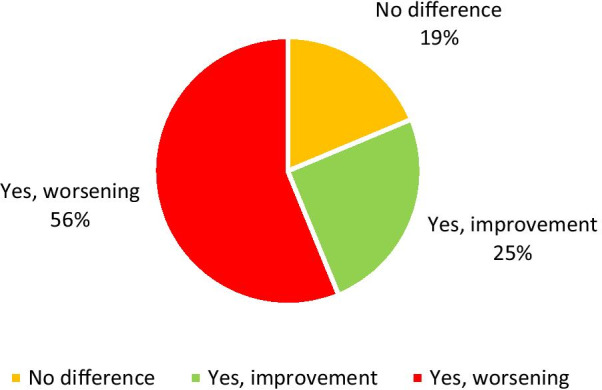
Student response rates regarding perceived changes in learning concentration during DE

More than 55% of the student sample (n = 114) reported significant impairment in concentration and learning abilities in attending online lessons, and surprisingly, a quarter reported experiencing better concentration and learning abilities (Fig. 2).

Among the students reporting the perceived concentration and learning impairment, 81.5% attributed the many or major difficulties of studying to a lack interaction with other colleagues, and 68.4% complained about completing DE in their own family environment, in which it was not easy to find their own quiet space (45.6%) (Fig. 1b).

Before home confinement, in the total sample, only 20.7% of the students were used to studying alone at their parents' homes. Approximately 80% were used to “*socially studying*” in different places such as flats rented with other students, university open spaces, or the university library, and they had to change their habits during pandemic.

**Fig. 3:**
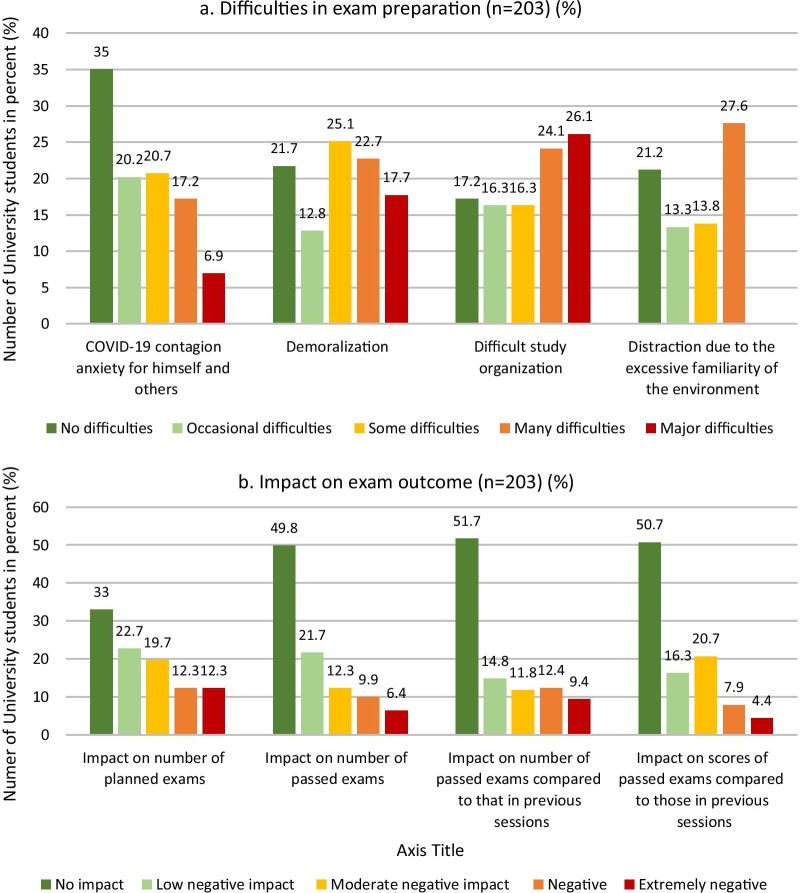
**a** Student response rates regarding difficulties preparing for exams during DE and **b** the impact of exam preparation difficulties on exam outcomes (n = 203)

The main difficulty in preparing for examinations during home confinement that the students reported seemed to be the excessive familiarity of the home environment, which distracted them (Fig. 3a). Surprisingly, COVID-19 contagion anxiety was the aspect that the lowest proportion of students worried about. Concerning academic performance and outcomes of the exams taken during DE, 24.6% of the students reported a negative or very negative impact on the number of scheduled exams, and 21.8% reported a negative or very negative impact on several exams, with the scores being significantly lower than those during the previous academic year. More than 15% had many difficulties or major difficulties passing exams, and 12.3% observed worsened exam results (Fig. 3b).

When asked about their perceived study performance, 71 students (35%) did not report any problems, 46 (27.7%) reported only occasional difficulties, 46 (27.7%) reported some difficulties, and 26 (12.8%) and 14 (6.9%) complained of many difficulties or major difficulties, respectively. When the last two groups were added to the analysis, approximately 20% (40 students) of the sample reported serious impairment of their academic performance.

**Fig. 4:**
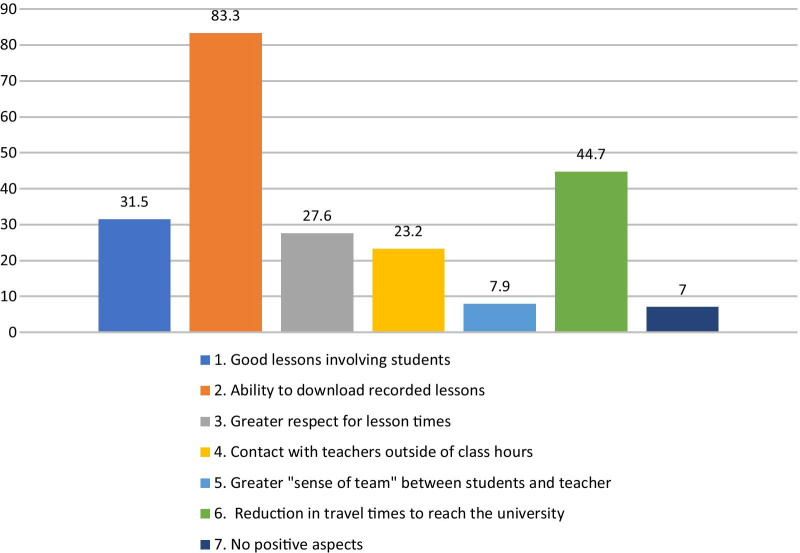
Advantages of distance education (n = 203)

Regarding the advantages of DE, 7% of the students did not observe any positive aspects. A total of 83.3% of the sample reported benefitting from teachers uploading online lessons to the platform, which allowed the lessons to be listened to again during exam preparation. The off-site students identified an advantage in the reduction in travel time, and one-third of students appreciated greater accuracy in carrying out online lessons by their teachers, who tried to take students' perspective to stimulate and motivate them (Fig. 4).

**Fig. 5:**
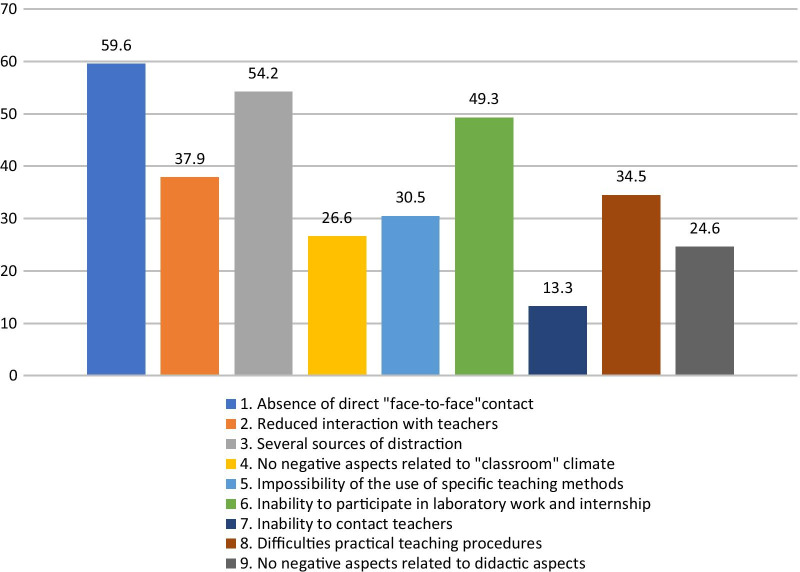
Disadvantages of distance education (%)—Classroom climate (bars 1–4) and didactic and organizational aspects (bars 5–9) (n = 203)

Concerning the disadvantages of DE, regarding the classroom climate, approximately 60% of the students reported the lack of “face-to-face” contact with teachers as the main negative aspect, and approximately 40% of them complained about difficulty interacting with teachers during the online lessons on the platform. More than 50% of the students considered the setting of DE, i.e., the family home, to be distracting. Regarding the students' responses about the didactic and organizational aspects of DE, approximately 50% of the sample complained about the lack of professional laboratory activity and internships. Approximately a quarter reported no negative aspects of DE (Fig. 5).

Concerning the students’ overall evaluations of DE on a scale from 1 to 10, the mean score was 6.65/10 (SD = 2.32), suggesting some degree of appreciation rather than neutrality. Regarding the students’ preferences for future teaching, 37% preferred completely remote teaching, 34.5% preferred mixed teaching, and 28.6% preferred face-to-face teaching.

The scores obtained for each item of our short version of the BDI-II are reported in Additional file 2. The BDI-II showed five main areas of impairment in our sample: sadness (reported by 79.3% of the total sample), changes in sleeping patterns (72.4%), lack of concentration (70.4%), loss of energy (69.5%), and pessimism (67%).

Regarding depressive symptoms as measured by the short version of the BDI, our sample had a mean score of 9.1 (SD = 5.9), indicating a moderate level of depression (according to the cut-off = 6 considered in this study). No statistically significant gender difference was found in the BDI-II total scores. Specifically, concerning the severity levels, the analyses carried out with Chi-square showed a statistically significant difference by gender, with a high proportion of women having a severe level of depression (chi-square: 8.813, d.f. 3; *p* = 0.032) (Table 2).

**Table 2:** Depression levels by gender

	Depression levels			
	None	Mild	Moderate	Severe
Gender				
Women	42	34	37_a_	42_a_
% of gender subgroup	27.1%	21.9%	23.9%	27.1%
Men	18	6	18	6
% of gender subgroup	37.5%	12.5%	37.5%	12.5%
Total	60	40	55	48
% Total	29.6%	19.7%	27.1%	23.6%

Concerning personal judgements of current health conditions, 69.5% (n = 141) of the sample reported having observed a slight-moderate improvement in their health, and 22.7% (n = 46) did not report any change. In comparison, 7.9% (n = 16) of students reported worse health conditions during the lockdown period than before the lockdown.

Regarding the Eyes Task scores used in our study to evaluate social cognition abilities, no statistically significant differences were observed among students showing different levels of depression. However, a statistically significant difference in the memory and concentration abilities measured by the five vignette stimuli taken from Module 5 of the MCT [38, 39] was observed. The analyses carried out with one-way ANOVA showed women more skilled than men in temporarily retaining non-verbal information (F = 3.964; *p* = 0.048) (Table 3).

**Table 3:** Psychopathological, memory and social cognition measures of the student sample by gender

	Student sample (n = 203)	Male students (n = 48)	Female students (n = 155)
BDI-II (range 0–30) mean (SD)	9.1 (5.9)	8 (5.7)	9.4 (5.9)
Memory and attention task (range 0–5) mean (SD)	1.09 (1.27)	0.77(0.9)	1.19 (1.34)*
Eyes task (range 0–10) mean (SD)	6.13 (1.6)	6.4 (1.4)	6.05 (1.6)

*p < 0.01

A total of 22.7% of students (n = 46) reported current relatively stable emotional well-being compared to that during the lockdown period, whereas 31.5% (n = 64) reported greater well-being, and 37.9% (n = 77) reported a slight improvement. Only 16 students (7.9%) expressed worsening of their emotional conditions.

### Correlations between the overall evaluation of the experience of DE and the variables included in the study

The correlations between the score assigned by students to the overall evaluation of the DE experience, the age of the students, scores on the BDI-II, on the Eyes Task, and the Memory Task are shown in Table 4.

**Table 4:** Correlations among the overall evaluation of distance education (DE) experience, age of students, BDI-II scores, Eyes Task scores, and Memory Task scores (n = 203)

		Overall evaluation of the DE experience	Age	BDI-II total score	Eyes task total score
Age	Pearson’s correlation	0.156*	–		
	2-Tailed *p* value	0.026			
BDI-II total score	Pearson’s correlation	− 0.359**	− 0.006	–	
	2-Tailed *p* value	0.000	0.935		
Eyes task total score	Pearson’s correlation	0.176*	0.152*	− 0.045	–
	2-Tailed *p* value	0.012	0.031	0.525	
Memory task total score	Pearson’s correlation	− 0.020	− 0.141*	− 0.035	− 0.074
	2-Tailed *p* value	0.782	0.045	0.627	0.294

**p < 0.01; *p < 0.05

The analyses carried out with correlation analysis (Pearson’s r) showed that the good overall evaluation of DE was positively correlated with the age of the students, the older ones displaying a better appreciation of the learning stimulated by this method, and with the social cognition ability assessed by the Eyes Task. Furthermore, the overall evaluation of the DE experience was significantly inversely correlated with BDI-II scores suggestive of depressive symptomatology, suggesting an association between a lower evaluation of DE, and a high expression of depressive symptoms.

### Predictors of academic performance during DE

The predictive model shown in Table 5 is the result of the logistic regression analysis to predict the students’ poor academic performance based on their subjective evaluations.

**Table 5:** Logistic regression analysis to predict poor academic performance based on the students’ self-assessments of their overall study performance during DE, divided into two categories: high (students reporting no problems, only occasional difficulties, or some difficulties) and low (many difficulties or major difficulties)

	Step 1					Step 2					Step 3				
	B	*p*	Exp (B)	CI 95% L	CI 95% U	B	*p*	Exp (B)	CI 95% L	CI 95% U	B	*p*	Exp (B)	CI 95% L	CI 95% U
*Gender*	0.428	0.285	1.535	0.700	3.365	0.488	0.236	1.630	0.727	3.655	1.145	**0.045**	**3.141**	1.026	9.615
Men = 0/Women = 1															
*Age* ≤ *25 years* = *1*	− 0.272	0.498	0.761	0.346	1.675	− 0.397	0.335	0.673	0.300	1.507	− 0.188	0.746	0.829	0.266	2.582
No = 0/Yes = 1															
*Working student*	− 0.350	0.498	0.705	0.256	1.939	− 0.347	0.509	0.707	0.253	1.977	− 0.249	0.724	0.779	0.196	3.102
No = 0/Yes = 1															
*Lack of sharing learning experience with other students*						− 0.081	0.843	0.922	0.414	2.056	0.313	0.572	1.367	0.462	4.050
No = 0/Yes = 1															
*Changes in the study context and habits following the pandemic*						1.366	**0.032**	**3.918**	1.124	13.659	0.053	0.946	1.055	0.228	4.884
No = 0/Yes = 1															
*Learning concentration impairment during distance education*											2.122	**0.014**	**8.350**	1.548	45.038
No = 0/Yes = 1															
*Eyes task inaccurate score*											0.408	0.720	1.504	0.162	13.996
Cut-off ≤ 3 = 1															
*Memory task total score*											− 0.364	0.089	0.695	0.456	1.058
*COVID-19 contagion anxiety*											1.213	**0.022**	**3.363**	1.190	9.506
No = 0/Yes = 1															
*10-item Beck Depression Inventory-II total score*											0.089	**0.047**	**1.093**	1.001	1.193
*Overall distance education evaluation score*											− 0.495	**0.000**	**0.610**	0.467	0.796

In the first step, among the main socio-demographic variables that were entered in the model, none showed statistically significant predictive power. In step 2, variables related to the DE experience, i.e., changes in study habits and the study environment following the pandemic, were entered; compared to students who were used to studying alone, those who were used to studying with other or in social environments had an approximately 4 times increased the likelihood of worse performance. Changes in the study context following the pandemic seemed to have significant predictive power in our model since we did not enter the cognitive, social cognition and psychopathological variables or the overall DE evaluation. This predictor was no longer statistically significant in the subsequent step when the individual impairment predictors were entered. In the third step, learning concentration impairment during DE showed significant predictive power, and the likelihood of students’ self-evaluation of their performance as poor increased by more than 8 times. High COVID-19 contagion anxiety for oneself and others increased the likelihood of poor academic performance by more than 3 times, and being a female student showed almost the same statistically significant risk. A high depressive symptomatology score, as measured by our short version of the BDI-II, seemed to contribute to a small increase in risk, just above 1 point.

In our sample, student satisfaction with DE seemed protective against poor outcomes; the overall distance education evaluation score O.R. of 0.10 (*p* = 0.000) implies that the satisfied group had almost half (60%) the odds of showing poor academic performance.

The Nagelkerke’s r2 values for the three blocks in the model in Table 5 were 0.015 for step 1, 0.062 for step 2, and 0.541 for step 3, providing an indication of the amount of variation in the dependent variable explained by the model.

## Discussion

The present study was designed to understand university students’ perspectives, attitudes, and readiness regarding online classes and investigate the impact of DE during the COVID-19 pandemic on their psychological health, social cognition, and memory function. Second, the study aimed to identify potential predictors of academic performance during the first Italian lockdown period.

As expected, we found a significant impact of distance education (DE), which was related to social, technological, and organizational adaptation difficulties, on students' psychological conditions, specifically, their depressive symptoms, and academic performance. DE was better appreciated by older students, displaying good social interaction abilities.

Recently, some studies have focused on the assessment of online class experience and overall online learning advantages and disadvantages [26, 27, 40, 41] and students’ psychological conditions [13, 22, 25, 42]. To the best of our knowledge, our study is the first to investigate the context and individual predictors of the impact of DE on academic outcomes in a sample of Italian students.

Regarding technical difficulties during delivery of DE, consistent with a recent study [29], our student sample mainly attributed having many difficulties to sharing a Wi-Fi network with other family members or friends and to network connection problems. It has been established that online learning cannot achieve the required intended learning outcomes if students do not have internet access due to technical or economic issues [43].

Regarding academic learning. Our data are only partially consistent with a recent study reporting that approximately 70% of students showed increased difficulties with virtual learning, primarily due to challenges with the available technology, unreliable internet connectivity, and perceived fatigue when listening to online lectures [41]. Interestingly, in our sample, a relevant factor related to learning ability was the study context. Before the COVID-19 home confinement, only approximately 20% of students were used to studying alone at their parents' homes, while 80% were used to “socially studying” in different places, such as flats rented with other students, in university open spaces, or the university library, and they had to change their habits during the pandemic.

Concerning academic performance and exams taken during DE, similar findings were observed in a recent study investigating academic achievements measured by students' grade point average (GPA) and curriculum objectives with remote E-exams. The study found that only 30% of students had higher GPAs, while approximately 40% reported no GPA change and approximately 30% had lower GPAs. Approximately 60% of all students reported that they did not achieve their curriculum objectives [44].

Regarding the advantages of DE, our findings are in line with a recent investigation conducted with a small student and teacher sample [27] that focused on positive and negative opinions about DE. The students seemed to have appreciated the possibility to take advantage of new academic resources (video lessons, etc.) and better and more autonomously manage their study activities. Similar findings were also observed in a larger sample that was more similar to ours in size [26]. The authors found a high appreciation for DE by just over 70% of the students, who liked studying through online classes since the study time became flexible and they could study anytime. Our sample of off-site students confirmed their appreciation of the reduction in travel time, and saving money and energy from using transportation was among the advantages of DE perceived by the students [40].

Among the disadvantages of DE, our findings confirmed that the absence of face-to-face interaction between students and teachers and the lack of traditional classroom socialization are among the issues of greatest interest in higher education [45, 46]. Our results are consistent with recent studies reporting the lack of the classroom environment [27], limited social contact [40], and lack of co-curricular activity [26].

Concerning the overall evaluation of DE on a scale from 1 to 10, the students in our sample expressed a mediocre level of appreciation, confirming the findings on the low appreciation of DE already reported in the literature; Sindiani et al. reported that 75% of the students in their study were not pleased with their experience of DE [40]. Conversely, while conventional learning was found to be more motivating than online learning by 70% of the students in one study [43], Shatakshi and Nardev [26] found that approximately 60% of students preferred the online continuity mode of teaching. The inconsistent appreciation of DE appears to be due to several factors that influence the overall DE experience, such as suboptimal or poor Internet connection or audio-visual media quality, unfamiliarity with completing online learning when it is suddenly implemented, and the lack of non-academic and social activities that make in-person education attractive to students [41].

Regarding depressive symptoms, as measured by the short version of the BDI, our sample showed a moderate level of depression. The young University students could feel severely distressed about the social isolation imposed due to COVID-19, in a phase of their life in which their peer group and interpersonal relationships have a significant impact on their emotional development and in establishing intimate relationships. The prevalence of medical students in our sample (more than 50%) students may have impacted the findings of our study. This student population is characterized by a competitive environment, a required continuous commitment, and a long academic journey. Many Authors showed among medical students an increased risk of depression compared to their peers currently enrolled in non-medical university courses [47, 48]. Italian surveys have found that medical students highlighted issues associated with anxiety and depression, emotional distress [49, 50], low perceived quality of life [51].

Specifically, for the severe level of depressive symptoms, a statistically significant difference was found by gender; a high proportion of women showed a severe depression level, which is consistent with recent studies [3, 21, 22]. The hypothesized reasons could be searched in differences in socialization processes and greater interpersonal sensitivity or sensitivity to the judgment of others of women [52, 53], differences in coping styles, emotion-oriented strategies for women and problem-oriented strategies for men [54].

Concerning social cognition abilities, no statistically significant differences were observed among students with different levels of depression. Our data are not in line with previous results showing that patients with major depressive disorder appear to decode emotions with a mood-congruent bias and have difficulty with cognitive theory of mind tasks requiring the interpretation of complex mental states [55], such as the Eyes Task used in our study. Our findings do not confirm that social cognitive performance is inversely associated with the severity of depression as reported by Weightman et al. [55].

Statistically significant differences in visual memory ability, measured by the five vignette stimuli taken from Module 5 of the MCT [38, 39], were observed, with higher scores for female students, who were more skilled than men in temporarily retaining non-verbal information. Our results do not seem to be in line with studies reporting no gender differences in visual memory abilities [56–58]. Our data agree with Feng et al., suggesting that women have a stronger cueing effect in the visual attention task, leading them to use more attention resources [59].

To achieve the second objective of the study, we investigated the variables that could predict poor academic performance during the provision of DE. The impact of DE on academic performance has been little explored, and the research has shown contradictory results. An Egyptian study did not find any statistically significant difference in students’ academic learning and performance in the shift from face-to-face to online DE due to the COVID-19 lockdown [60]. A significant positive effect of COVID-19 confinement on students’ performance was reported by Gonzalez et al. [42], who showed that students’ learning strategies became more continuous habits, improving their efficiency and autonomous learning performance. The authors attributed this improvement to students facing a new scenario; they were afraid of missing the academic year because of the COVID-19 confinement, and they worked harder to overcome any difficulty, motivated by their intrinsic responsibility in a perplexing situation [42]. In the USA, the impact of COVID-19 on student experiences and expectations seemed to include delayed graduation; loss of jobs, internships, and job offers; and expectations of earning less at age 35 than originally anticipated. These impacts were mainly attributed to existing socioeconomic divides, with lower-income students 55% being more likely than their higher-income peers to have delayed graduation due to COVID-19 [61].

In our first 2 logistic regression analysis steps, gender, age, working student status, and lack of sharing learning experience with other students did not show any predictive value for low academic performance. The only significant predictor was changes in the study environment, which increased the likelihood of students' poor academic performance by almost 4 times. The students were used to living in a flat with their colleagues, studying with other students, and/or studying “in social settings” but then had to come back to their parents' homes and stay home all day. Inconsistent with our findings, a third of the sample of the study by Shatakshi and Nardev seemed to greatly appreciate experiencing “the study location as flexible” and, probably, for them, more functional [26]. We hypothesize that several factors could be the basis of such academic impairment, such as the drastic reduction in outings and social interactions, as described in Elmer et al. [13], the reduction in personal autonomy, and minor family conflicts in the restricted home area. In our sample, living with parents did not seem to be a protective factor [17].

The addition of individual psychological and psychopathological variables to the context variables related to the stressful condition of home confinement enriched our “risk model”: change in the study context and study habits was no longer statistically significant in our third analysis step, and the strongest predictor was concentration impairment, which increased the likelihood of poor academic performance by more than 8 times. Reduction in concentration during the pandemic lockdown in university students has been observed in several studies [22, 25, 62]. Our study was the first to identify its specific role as a strong predictor of poor academic performance.

The likelihood of poor academic performance seemed to increase by more than 3 times in students presenting COVID-19 contagion anxiety for themselves or others. This study result is partially consistent with a study conducted in the United Arab Emirates, showing that anxiety about COVID-19 contagion significantly increased the likelihood of university students' psychological distress by almost 3 times [63]. We did not find a statistically significant gender difference in COVID-19 contagion anxiety; this results is inconsistent with that of Rodriguez et al., who reported that women showed higher fear of COVID-19 in their sample of Ecuadorian university students [64]. However, in our regression model, being a woman was a statistically significant predictor of poor academic performance, as was depressive symptomatology, albeit with less predictive power. Our data indirectly support the finding that women perceive remote E-exams to be more stressful than male respondents [44], and our female sample showed a more severe depression level and more than 3 times the likelihood of poor academic performance than their male colleagues.

Finally, it was not surprising that good appreciation could represent a protective factor against academic failure, suggesting that the acceptance of this new learning modality could underline relevant cognitive flexibility and global well-being, as shown in a previous study [22].

### Strengths and limitations

Learning concentration impairment, COVID-19 contagion anxiety, female gender, and depressive symptomatology were identified as predictors of poor academic performance in a sample of university students during home confinement and online learning. This is the main strength of this first Italian study on this topic, which investigates a comprehensive risk model, as well as technological and context aspects related to DE and psychopathological, social cognitive, and cognitive variables.

Some limitations of the present study should be acknowledged. First, the study was based on a cross-sectional online anonymous survey using a convenience sample. Second, the study was conducted among a sample from a single academic department, and the sample size (of both participants and setting) represents a further limitation for generalizability. The small sample size could be attributed mainly to the selected period (July 15–September 30), including exams preparation, graduations, and summer holidays time. Indeed, we were interested to investigate the students' condition before the start of the new academic year and relatively close to the more strict lockdown period, identifying a fatigue scenario due to Covid-19 social restrictions. Third, no validated measures were used in our study in the absence of international validated instruments assessing DE. The “Studying with COVID” survey was developed by a focus group of experienced professionals working in a counselling and consultation university service, teachers and senior students based on their suggested main themes. Four, a more comprehensive battery for psychopathological, social cognition and memory function assessments would have been useful for better characterizing our student sample. Our short version of the BDI reflects the authors’ choice to be “less invasive” (items on suicidal ideation, sexuality, etc., were omitted) and to “normalize” the sort of post-traumatic reaction expected to follow COVID-19 social restriction measures. Based on some of the authors’ experience working on the topic of post-traumatic distress [65, 66], within certain limits, distress is perfectly natural and normal in a context such as the COVID-19 pandemic. Additionally, the use of a short-digitized version of the Eyes Task and the five vignette stimuli taken from Module 5, “Overconfidence in memory errors,” have not been validated for the mode of administration used in this study, which was the only possible mode in the contingent emergency context.

## Conclusions

The COVID-19 outbreak inevitably had a significant impact on university students' lives and habits, acting as a cause of difficulty and suffering.

The study confirms the emerging need for monitoring and work on “modifiable” risk factors for poor academic performance related to DE, which occurred during the 2019/2020 academic year and will continue in the coming months, to meet psychological students' needs. The objectives are to ensure the continuation of the educational relationship between teachers and students, students’ psychological well-being, even during the COVID-19 pandemic, and their study success.

University counselling services could play a fundamental role in supporting and helping students who are faced with emotional-psychological distress during their studies and in difficult moments, such as the current health emergency, focusing on individual vulnerabilities.

## Supplementary Information

**Additional file 1.** Survey instruments and related measures.

**Additional file 2.** The scores obtained for each item of our short version of the BDI-II are reported.

## Data Availability

The data sets used and analysed during the current study are available from the corresponding author on reasonable request.
