# Supplementary material for: Predictors of academic performance during the covid-19 outbreak: impact of distance education on mental health, social cognition and memory abilities in an Italian university student sample
Source: BMC Psychol. 2021 Sep 15;9:142. doi: 10.1186/s40359-021-00649-9 (PMC8441245; doi:10.1186/s40359-021-00649-9)
Supplement: Supplementary file 1 — Additional file 1. Survey instruments and related measures. [file 40359_2021_649_MOESM1_ESM.pdf]

# QUESTIONNAIRE ON STUDENT PERSPECTIVES OF DISTANCE EDUCATION (DE)

## SECTION 1. INFORMATION ON THE STUDY, PROTECTION OF PRIVACY, AND INFORMED CONSENT

## SECTION 2. DISTANCE EDUCATION FROM THE STUDENT PERSPECTIVE

### 2.1 MAIN DEMOGRAPHIC AND ACADEMIC DATA

**1. Sex**

- ❒ Man
- ❒ Woman

**2. Age [__] [__]**

**3. Academic program:**

- ❒ Medicine and Surgery
- ❒ Dentistry and Dental Prosthetics
- ❒ Nursing and Obstetric Sciences
- ❒ Sciences of Technical Health Professions
- ❒ Sciences of Prevention Health Professions
- ❒ Nursing and Obstetric Sciences
- ❒ Speech Therapy
- ❒ Obstetrics
- ❒ Orthoptics and Ophthalmological Assistance
- ❒ Psychiatric Rehabilitation Technique
- ❒ Neuro and Psychomotor Childhood Therapy
- ❒ Dental Hygiene
- ❒ Techniques of Prevention in the Environment and in the Workplace
- ❒ Biology of Health and Nutrition
- ❒ Biological Sciences
- ❒ Environmental Biology and Ecosystem Management
- ❒ Environmental Sciences and Technologies

**5. What kind of student are you?**

- ❒ Full-time current student
- ❒ Part-time current student
- ❒ Off-course student

**6. In the 2019-20 A.Y., in which year of the program are you enrolled?**

- ❒ 1st year
- ❒ 2nd year
- ❒ 3rd year
- ❒ 4th year
- ❒ 5th year
- ❒ 6th year
- ❒ Off-course student

**7. Are you an off-site student?**

- ❒ No
- ❒ Yes

**8. Are you a working student?**

- ❒ No
- ❒ Yes

**9. In the second semester in the 2019-20 A.Y., did you have remote exams?**

- ❒ Yes
- ❒ No

## Section 2.2 DISTANCE EDUCATION EXPERIENCES

### 2.2.1 TECHNOLOGICAL ASPECTS

**How was your experience concerning the TECHNOLOGICAL ASPECTS of DE in the second semester of the 2019-2020 A.Y.?**

| | No difficulty | Occasional difficulties | Some difficulty | Many difficulties | Major difficulties |
|---|---|---|---|---|---|
| **9.** Connection quality | | | | | |
| **10.** Device availability | | | | | |
| **11.** Use of the Teams platform | | | | | |
| **12.** Other | | | | | |

**13. In your home, have you had problems with family overlap in the use of the WiFi network and hardware?**

- ❒ No problems
- ❒ Minor inconveniences, without significant consequences
- ❒ Significant inconvenience and frequent loss of effectiveness of the connection and/or hardware sharing
- ❒ Frequent inability to connect due to network or hardware sharing
- ❒ Other (specify …………………………………… )

### 2.2.2 ACADEMIC LEARNING ASPECT

**14. Do you think DE influenced your ability to follow lessons, concentrate, and learn?**

- ❒ No, I didn't notice any difference compared to classroom lessons.
- ❒ Yes, I noticed some improvements. I felt much more alert and focused on my tasks.
- ❒ Yes, I have noticed a deterioration in my abilities.

**If you observed worsening in following your DE lessons, what do you think could be the reasons?**

| | Major difficulties | Many difficulties | Some difficulty | Occasional difficulties | No difficulty |
|---|---|---|---|---|---|
| **15.** More easily distracted due to the excessive familiarity of the environment that did not create a stimulus to learn | | | | | |
| **16.** The awareness that the lessons were recorded and therefore could be reviewed | | | | | |
| **17.** Lack of an isolated and quiet environment in the house | | | | | |
| **18.** Lack of interaction and sharing experience with other students | | | | | |

**19. Changes in study setting and habits during the COVID-19 pandemic:**

- ❒ I did not change my habits since I was studying on my own at my parents' home both before and after the pandemic.
- ❒ I had to change my habits since I was studying in my rented flat with other students on my own and/or with other colleagues before pandemic.
- ❒ I had to change my habits since I was studying on my own in the university library before pandemic.
- ❒ I had to change my habits since I was studying in dedicated spaces at the university dedicated spaces on my own and/or with other colleagues before pandemic.

**Regarding exam preparation during lockdown:**

| | Major difficulties | Many difficulties | Some difficulty | Occasional difficulties | No difficulty |
|---|---|---|---|---|---|
| **20.** Did you find it more difficult to concentrate and to adhere to the daily study program due to the fear of COVID-19 contagion for yourself or others? | | | | | |
| **21.** Did you find it more difficult to concentrate and to adhere to the daily study program due to a state of demoralization? | | | | | |
| **22.** Did you find it more difficult to organize your study despite having more available time? | | | | | |
| **23.** Have you found a greater tendency to be distracted by an overly familiar environment? | | | | | |

**Impact on exam outcomes**

| | Extremely negative | Negative | Moderate negative impact | Low negative impact | No impact |
|---|---|---|---|---|---|
| **24.** Impact on the scheduled number of exams in the April and summer sessions | | | | | |
| **25.** Impact on the number of passed exams | | | | | |
| **26.** Impact on the number of passed exams compared to that in previous sessions | | | | | |
| **27.** Impact on the scores of passed exams compared to those in previous sessions | | | | | |

**Self-assessment of overall study performance during DE**

| | Major difficulties | Many difficulties | Some difficulty | Occasional difficulties | No difficulty |
|---|---|---|---|---|---|
| **28.** Overall, what is your assessment of your academic performance during DE? | | | | | |

### 2.2.3 ADVANTAGES OF DISTANCE EDUCATION

**29. What advantages of DE did you notice?**

- ❒ Greater care in adapting the lessons to stimulate the students and involve them through active participation
- ❒ Opportunity to download recorded lessons
- ❒ Greater respect for lesson times
- ❒ Greater opportunity to contact teachers outside class hours for clarification via email, WhatsApp, etc.
- ❒ Greater sense of team between students and teachers
- ❒ Reduction in the travel time necessary to reach the university
- ❒ I honestly didn't find any positive aspects
- ❒ Other (specify……………………………………………………….. )

### 2.2.4 DISADVANTAGES OF DISTANCE EDUCATION

**30. What negative aspects of DE did you notice (classroom climate)?:**

- ❒ Absence of direct "face-to-face" eye contact with professors
- ❒ Reduced interaction with professors during lessons
- ❒ Distracting effect due to the study environment
- ❒ I didn't find any negative aspect
- ❒ All the above

**31. What negative aspect of DE did you notice (didactic and organizational aspects)?**

- ❒ Lack of specific teaching methods (e.g., role playing)
- ❒ Absence of laboratory professionalizing activities and internship
- ❒ Difficulty of reaching teachers
- ❒ Difficulties related to carrying out small administrative procedures related to didactics (e.g., AFO)
- ❒ I didn't find any negative aspect
- ❒ All the above

### 2.2.5 OVERALL EVALUATION OF THE DE EXPERIENCE and SUGGESTIONS

**32. What kind of teaching would you recommend?**

- ❒ I would like face-to-face teaching
- ❒ I would like mixed teaching
- ❒ I would like completely remote teaching

**33. How would you evaluate your overall DE experience (1 = very negative; 10 = excellent)? [_][_]**

**Comments and suggestions**

…………………………………………………………………………………………………………………………………………………………

…………………………………………………………………………………………………………………………………………………………

…………………………………………………………………………………………………………………………………………………………

**STUDENTS' EMOTIONAL WELL-BEING DURING THE COVID-19 LOCKDOWN**

*In the two months of confinement due to COVID-19, it is possible that, as a person, you have experienced moments of difficulty and emotional distress. We would be grateful if you could answer the questions presented to you below, which will help us understand how much your state has influenced any perception of difficulty and dissatisfaction, even in your role as a student. In answering the questions, we invite you to refer to the 2 months of confinement (mid-March - early May 2020).*

**Sadness**
0. I did not feel sad
1. I felt sad
2. I was sad all the time, and I couldn't snap out of it
3. I was so sad or unhappy that I couldn't stand it

**Pessimism**
0. I was not particularly discouraged about the future
1. I felt discouraged about the future
2. I felt I had nothing to look forward to
3. I felt the future was hopeless and that things could not improve

**Loss of pleasure**

0. I got as much satisfaction out of things as I used to
1. I didn't enjoy things the way I used to
2. I didn't get real satisfaction out of anything anymore
3. I was dissatisfied or bored with everything

**Loss of interest**
0. I didn't lose interest in other people or activities
1. I was less interested in other people or things than before
2. I lost most of my interest in other people or things
3. It was hard to get interested in anything

**Loss of energy**
0. I had as much energy as ever
1. I had less energy than I used to have
2. I didn't have enough energy to do very much
3. I didn't have enough energy to do anything

**Changes in sleeping pattern**
0. I did not experience any change in my sleeping pattern
1.a I slept somewhat more than usual
1.b I slept somewhat less than usual
2.a I slept a lot more than usual
2.b I slept a lot less than usual
3.a I slept most of the day
3.b I woke up 1-2 hours early, and I couldn't get back to sleep

**Irritability**
0. I was not more irritable than usual
1. I was more irritable than usual
2. I was much more irritable than usual
3. I was irritable all the time

**Changes in appetite**

0. I did not experience any change in my appetite
1.a My appetite was somewhat less than usual
1.b My appetite was somewhat greater than usual
2.a My appetite was much less than usual
2.b My appetite was much greater than usual
3.a I had no appetite at all
3.b I craved food all the time

**Concentration difficulty**

0. I could concentrate as well as ever
1. I couldn’t concentrate as well as usual
2. It was hard to keep my mind on anything for very long
3. I found I couldn't concentrate on anything

**Tiredness or fatigue**

0. I was no more tired or fatigued than usual
1. I got tired or fatigued more easily than usual
2. I was too tired or fatigued to do a lot of the things I used to do
3. I was too tired or fatigued to do most of the things I used to do

***Compared to the beginning of last May (end of confinement), how would you judge your emotional condition now?***

5 = much better now
4 = a little better now
3 = more or less the same
2 = a little worse now
1 = much worse now

**SPEAK WITH YOUR EYES DESPITE THE MASKS**

Below, you will be presented with 10 slides, with each one showing a photograph representing the eye region of a male or female subject's face. Under each photograph, four mental states can be associated with the represented gaze. Your task is to carefully observe the photograph and choose the option corresponding to the most appropriate mood.

**Image 1**

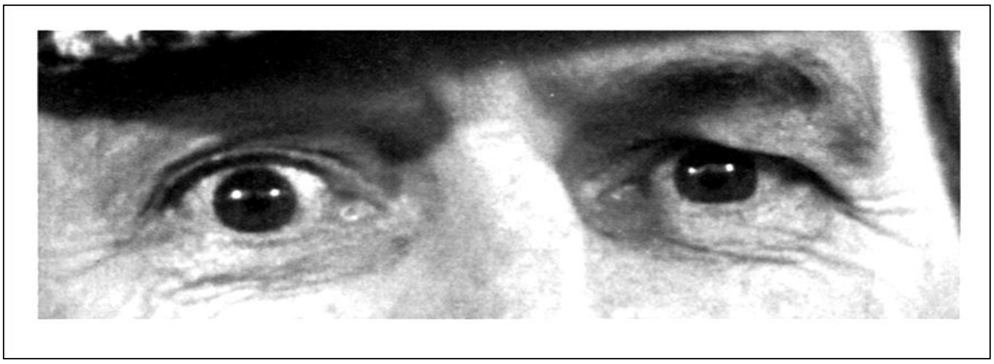

- jealous
- panicked
- arrogant
- hateful

**Image 2**

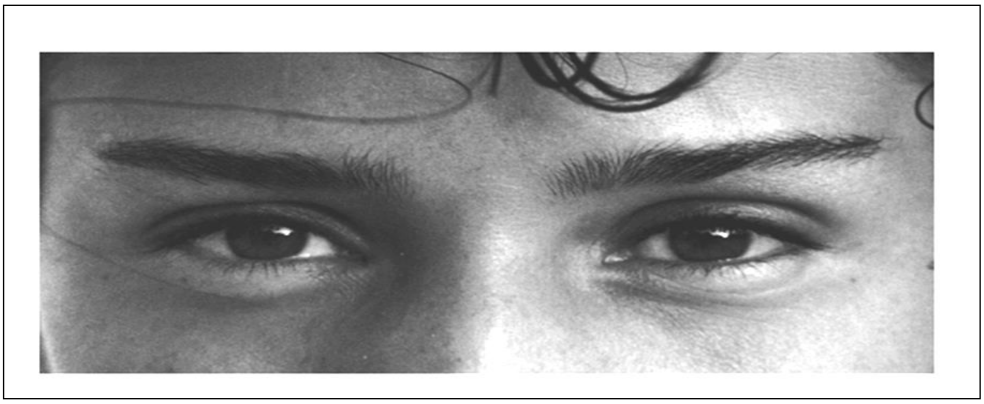

- playful
- comforting
- irritated
- bored

**Image 3**

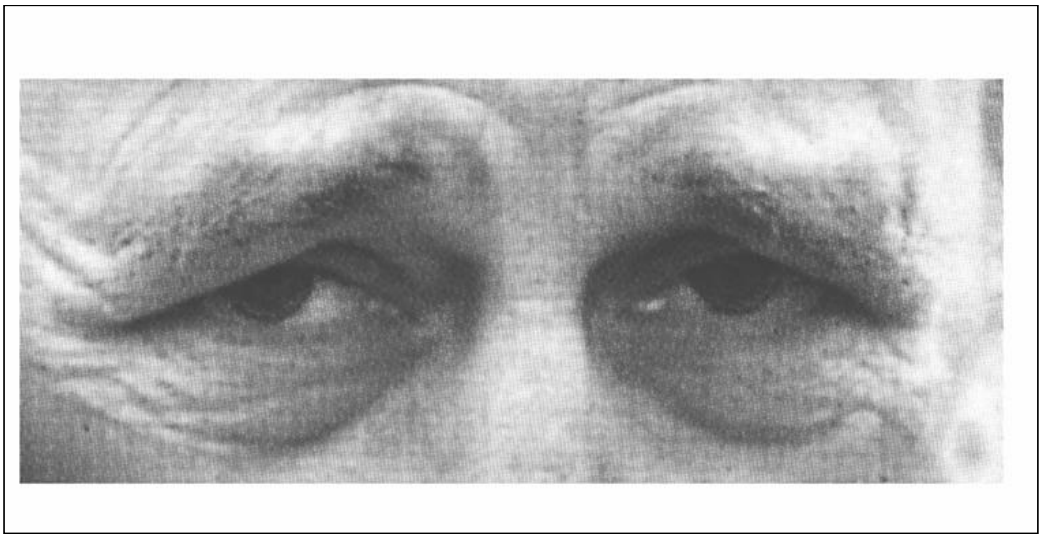

- terrified
- upset
- arrogant
- annoyed

**image 4**

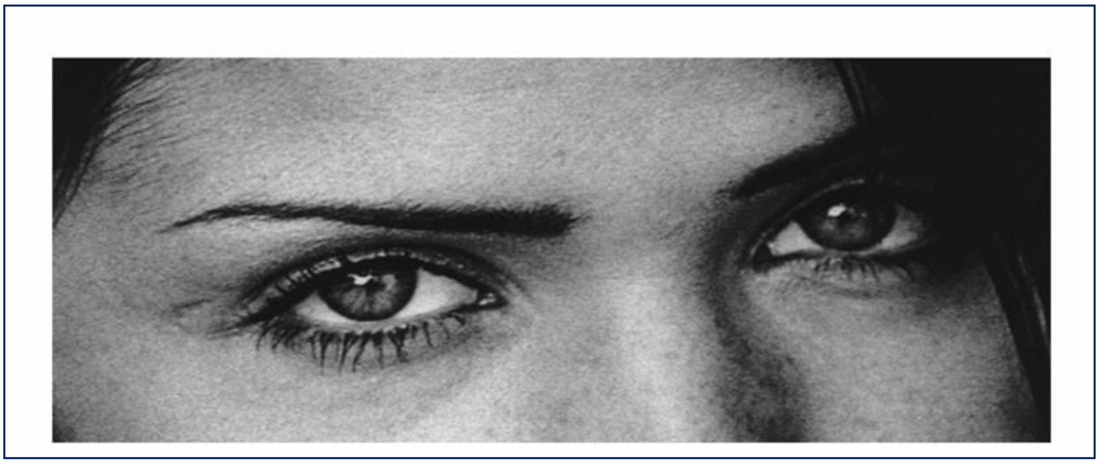

- joking
- flustered
- desirous
- convinced

**Image 5**

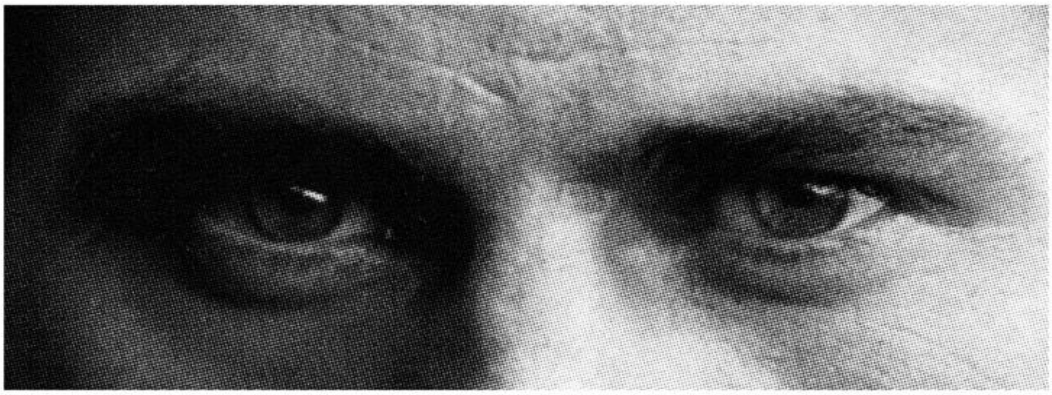

- joking
- insisting
- amused
- relaxed

**image 6**

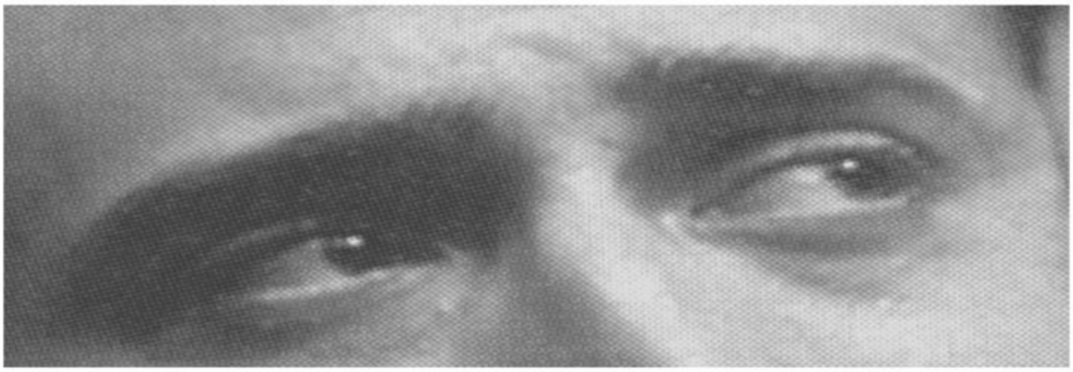

- irritated
- sarcastic
- worried
- friendly

**image 7**

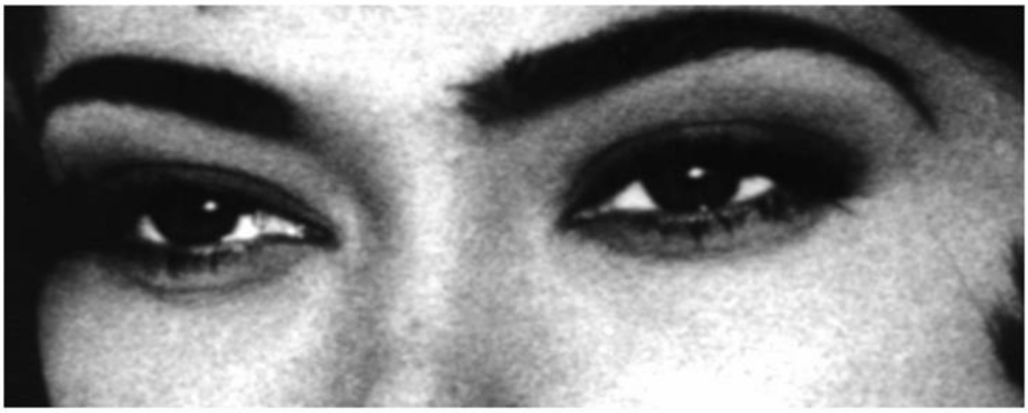

- aghast
- fantasizing
- impatient
- alarmed

**image 8**

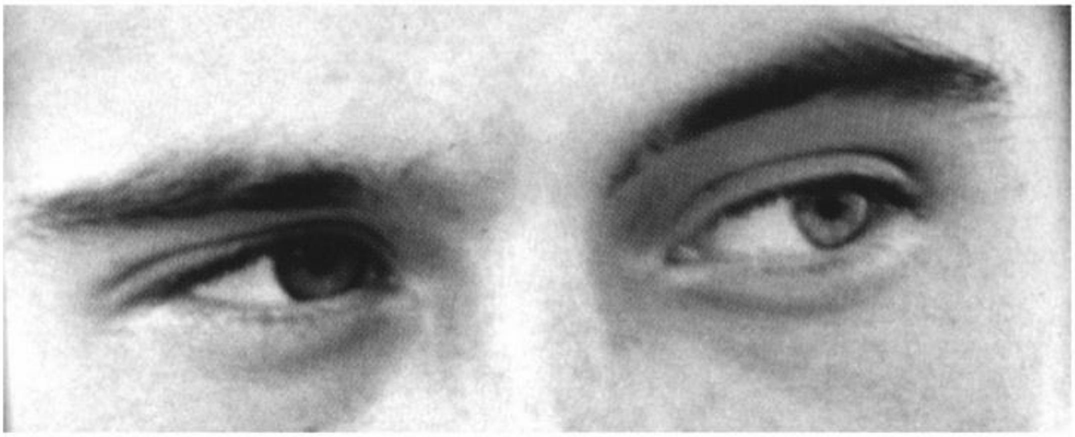

- apologetic
- friendly
- uneasy
- dispirited

**image 9**

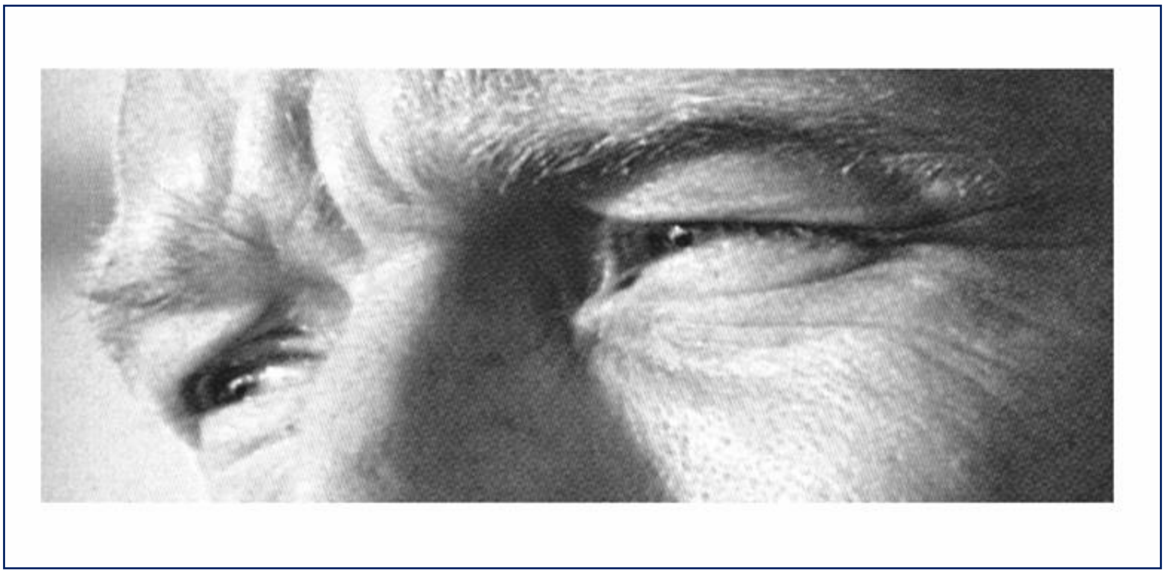

- despondent
- relieved
- shy
- excited

**image 10**

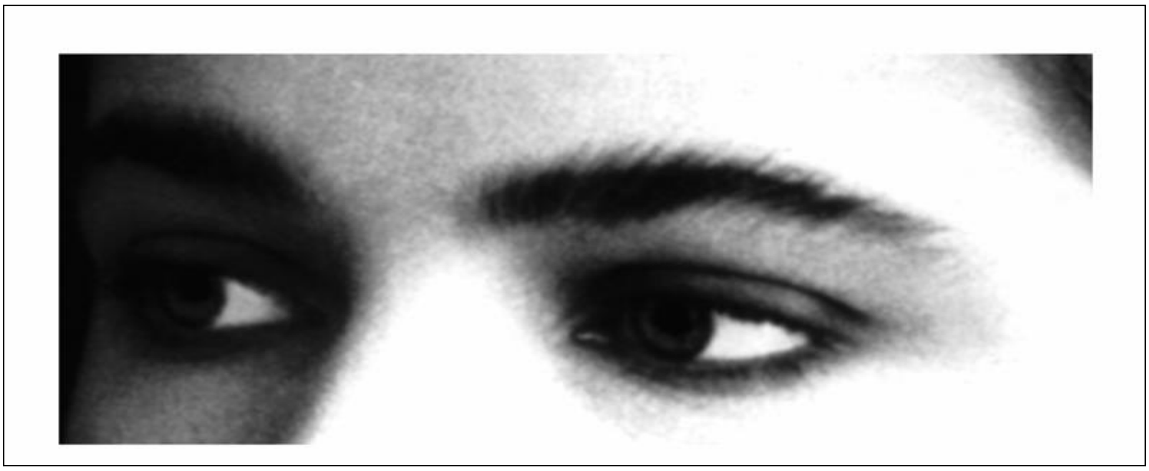

- annoyed
- hostile
- horrified
- preoccupied

**MIND THE WIND!**

Let's do a test to verify your attention with some images, on which we ask you to not linger for more than 10 seconds.

**Image 1.** What does this image make you think about? What does it remind you of?

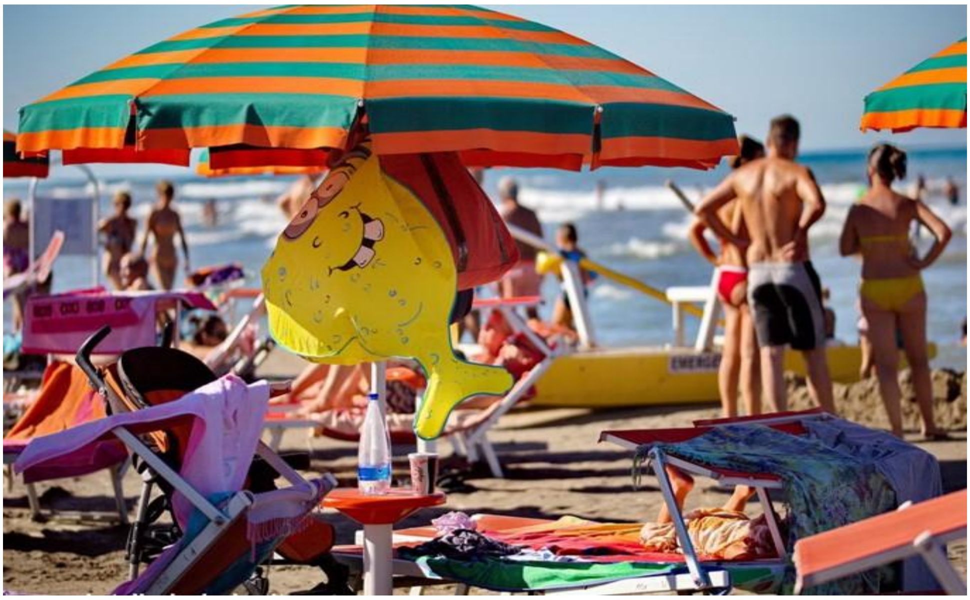

- It is a lively popular image that makes you want to go to the beach
- I like deserted beaches
- Other

**Question 1. screen**

Try to remember the previous image. Which of the following elements were missing?

- Ball
- Umbrella
- Hat
- Deckchair
- Boat
- Beach bag
- Water
- Beach towel

**Image 2.** What does this image make you think about? What does it remind you of?

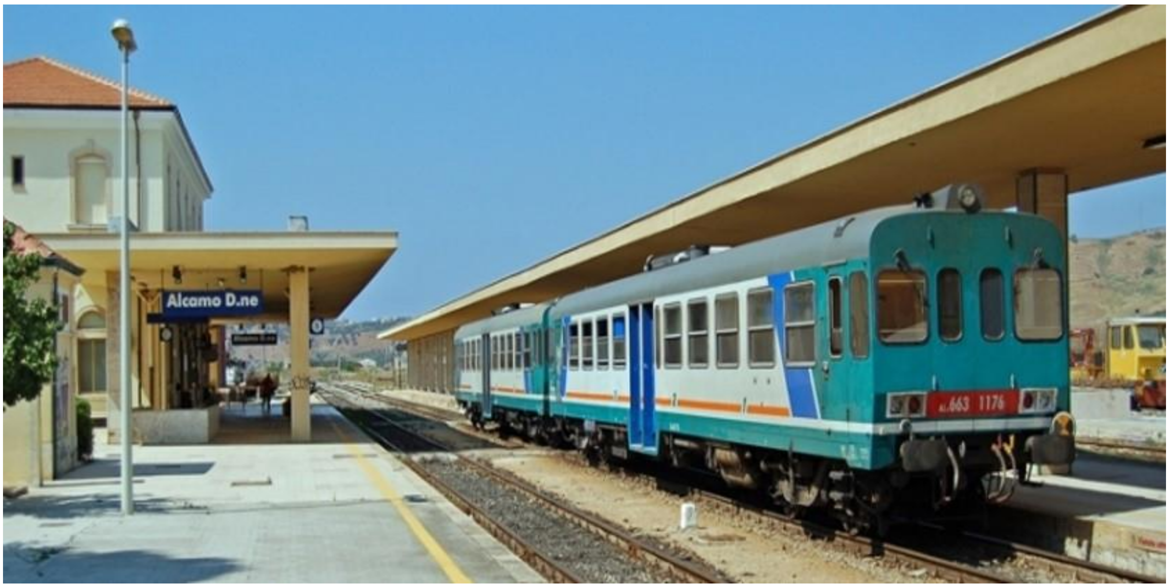


- It's a familiar picture
- It’s an old picture
- Other

**Question 2. screen**

Try to remember the previous image. Which of the following elements were missing?

- Clock
- Rails
- Trolley
- Carriage
- Travelers
- Railway signal
- Streetlamp
- Driving force

**Image 3.** What does this image make you think about? What does it remind you of?

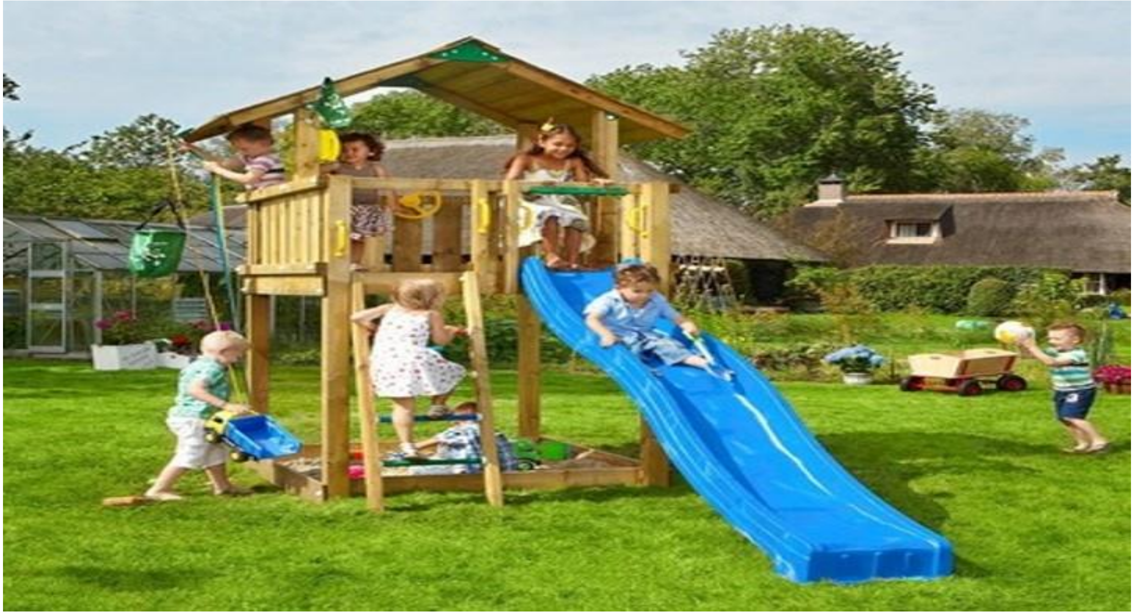

- My playground when I was child
- My park was different
- Other

**Question 3. screen**

Try to remember the previous image. Which of the following elements were missing?

- Swing
- Children
- Trees
- Wooden ladder
- Kite
- Pickup truck
- Ball
- Slide

**Image 4.** What does this image make you think about? What does it remind you of?

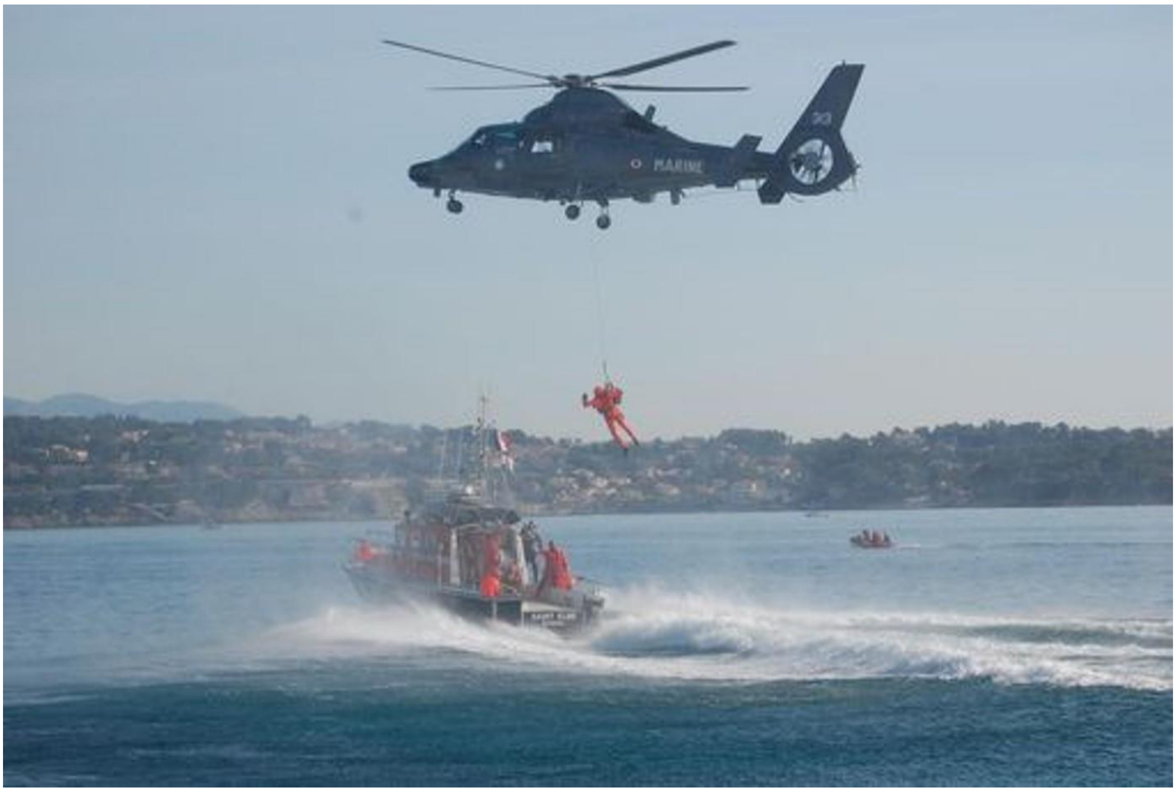

- It's a scene from a movie
- It is the scene of an authentic rescue
- Other

**Question 4. screen**

Try to remember the previous image. Which of the following elements were missing?

- Stretcher
- Man tied to a rope
- Life buoy
- Landscape
- Divers
- Water
- Helicopter
- Boat

**Image 5.** What does this image make you think about? What does it remind you of?

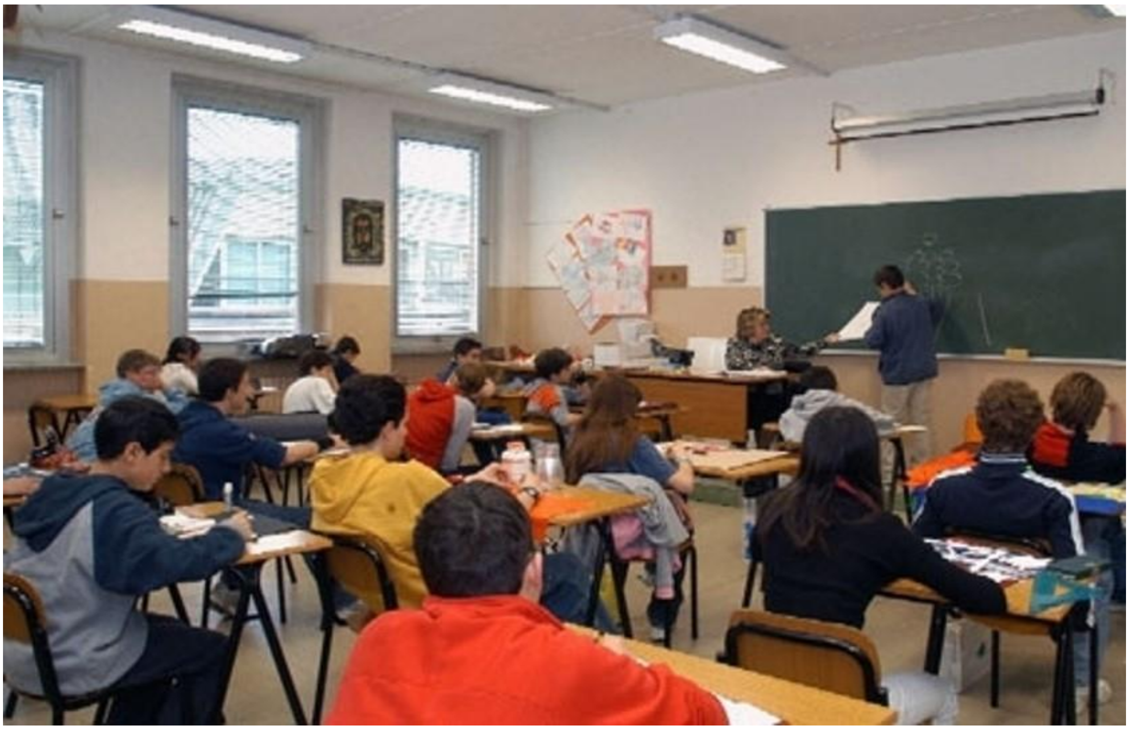

- A class in my adolescence
- The questions on the blackboard were a nightmare!
- Other

**Question 5. screen**

Try to remember the previous image. Which of the following elements were missing?

- Chair
- Windows
- Backpack
- Teacher
- Teacher's desk
- Geographic map
- Blackboard
- Books

*Thank you for your collaboration*
