# Supplementary material for: Predictors of academic performance during the covid-19 outbreak: impact of distance education on mental health, social cognition and memory abilities in an Italian university student sample
Source: BMC Psychol. 2021 Sep 15;9:142. doi: 10.1186/s40359-021-00649-9 (PMC8441245; doi:10.1186/s40359-021-00649-9)
Supplement: Supplementary file 2 — Additional file 2. The scores obtained for each item of our short version of the BDI-II are reported. [file 40359_2021_649_MOESM2_ESM.pdf]

SUPPLEMENTARY MATERIAL

Depressive symptoms measured by 10 items derived from the BDI-II

	Total (%)
**Sadness**	
0. I did not feel sad	42 (20.7)
1. I felt sad	117 (57.6)
2. I was sad all the time, and I couldn't snap out of it	32 (15.8)
3. I was so sad or unhappy that I couldn't stand it	11 (5.4)
Missing	1 (0.5)
**Pessimism**	
0. I was not particularly discouraged about the future	67 (33)
1. I felt discouraged about the future	99 (48.8)
2. I felt I had nothing to look forward to	22 (10.8)
3. I felt the future was hopeless and that things could not improve	14 (6.9)
Missing	1 (0.5)
**Loss of pleasure**	
0. I got as much satisfaction out of things as I used to	99 (48.8)
1. I didn’t enjoy things the way I used to	48 (23.6)
2. I didn’t get real satisfaction out of anything anymore	42 (20.7)
3. I was dissatisfied or bored with everything	13 (6.4)
Missing	1 (0.5)
**Loss of interest**	
0. I didn't lose interest in other people or activities	96 (47.3)
1. I was less interested in other people or things than before	63 (31)
2. I lost most of my interest in other people or things	31 (15.3)
3. It was hard to get interested in anything	11 (5.4)
Missing	2 (1)
**Loss of energy**	
0. I had as much energy as ever	62 (30.5)
1. I had less energy than I used to have	100 (49.3)
2. I didn't have enough energy to do very much	32 (15.8)
3. I didn't have enough energy to do anything	8 (3.9)
Missing	1 (0,5)
**Changes in sleeping pattern**	
0. I did not experience any change in my sleeping pattern	56 (27.6)
1.a I slept somewhat more than usual	61 (30)
1.b I slept somewhat less than usual	41 (20.2)
2.a I slept a lot more than usual	16 (8)
2.b I slept a lot less than usual	19 (9.4)
3.a I slept most of the day	1 (0.5)
3.b I woke up 1-2 hours early, and I couldn’t get back to sleep	9 (4.4)
Missing	
**Irritability %**	
0. I was not more irritable than usual	74 (36.5)
1. I was more irritable than usual	78 (38.4)
2. I was much more irritable than usual	36 (17.7)
3. I was irritable all the time	13 (6.4)
Missing	2 (1)

**Changes in appetite %**	
0. I did not experience any change in my appetite	78 (38.4)
1.a My appetite was somewhat less than usual	25 (12.3)
1.b My appetite was somewhat greater than usual	71 (35)
2.a My appetite was much less than usual	9 (4.4)
2.b My appetite was much greater than usual	19 (9.4)
3.a I had no appetite at all	-
3.b I craved food all the time	-
Missing	1 (0.5)
**Concentration difficulty %**	
0. I could concentrate as well as ever	60 (29.6)
1. I couldn’t concentrate as well as usual	76 (37.4)
2.It was hard to keep my mind on anything for very long	55 (27.1)
3. I found I couldn't concentrate on anything	11 (5.4)
Missing	1 (0.5)
**Tiredness or fatigue %**	
0. I was no more tired or fatigued than usual	87(43)
1. I got tired or fatigued more easily than usual	85 (42)
2. I was too tired or fatigued to do a lot of the things I used to do	28 (13.8)
3. I was too tired or fatigued to do most of the things I used to do	3 (1.5)
Missing	
